# Human amnion-derived mesenchymal stem cell (hAD-MSC) transplantation improves ovarian function in rats with premature ovarian insufficiency (POI) at least partly through a paracrine mechanism

**DOI:** 10.1186/s13287-019-1136-x

**Published:** 2019-01-25

**Authors:** Li Ling, Xiushan Feng, Tianqin Wei, Yan Wang, Yaping Wang, Ziling Wang, Dongyuan Tang, Yanjing Luo, Zhengai Xiong

**Affiliations:** 1 Department of Obstetrics and Gynecology, The Second Affiliated Hospital of Chongqing Medical University, No. 76, Linjiang Road, Chongqing, 400010 China; 2 State Key Laboratory of Ultrasound Engineering in Medicine Co-Founded by Chongqing and the Ministry of Science and Technology, Chongqing Key Laboratory of Biomedical Engineering, College of Biomedical Engineering, Chongqing Medical University, Chongqing, 400010 China; 3 Department of Histology and Embryology, Laboratory of Stem Cell and Tissue Engineering, Chongqing Medical University, Chongqing, 400010 China

**Keywords:** Premature ovarian insufficiency (POI), Human amnion-derived mesenchymal stem cells (hAD-MSCs), Paracrine, Conditioned media (CM), Growth factors

## Abstract

**Background:**

Chemotherapy can induce premature ovarian insufficiency (POI) and reduce fertility in young female patients. Currently, there is no effective therapy for POI. Human amnion-derived mesenchymal stem cells (hAD-MSCs) may be a promising *seed cell* for regenerative medicine. This study investigated the effects and mechanisms of hAD-MSC transplantation on chemotherapy-induced POI in rats.

**Methods:**

Chemotherapy-induced POI rat models were established by intraperitoneal injection of cyclophosphamide. Seventy-two female SD rats were randomly divided into control, POI, and hAD-MSC-treated groups. hAD-MSCs were labeled with PKH26 and injected into the tail veins of POI rats. To examine the underlying mechanisms, the differentiation of transplanted hAD-MSCs in the POI ovaries was analyzed by immunofluorescent staining. The in vitro expression of growth factors secreted by hAD-MSCs in hAD-MSC-conditioned media (hAD-MSC-CM) was analyzed by ELISA. Sixty female SD rats were divided into control, POI, and hAD-MSC-CM-treated groups, and hAD-MSC-CM was injected into the bilateral ovaries of POI rats. After hAD-MSC transplantation or hAD-MSC-CM injection, serum sex hormone levels, estrous cycles, ovarian pathological changes, follicle counts, granulosa cell (GC) apoptosis, and Bcl-2, Bax, and VEGF expression in ovaries were examined.

**Results:**

PKH26-labeled hAD-MSCs mainly homed to ovaries after transplantation.

hAD-MSC transplantation reduced ovarian injury and improved ovarian function in rats with POI. Transplanted hAD-MSCs were only located in the interstitium of ovaries, rather than in follicles, and did not express the typical markers of oocytes and GCs, which are ZP3 and FSHR, respectively. hAD-MSCs secreted FGF2, IGF-1, HGF, and VEGF, and those growth factors were detected in the hAD-MSC-CM. hAD-MSC-CM injection improved the local microenvironment of POI ovaries, leading to a decrease in Bax expression and an increase in Bcl-2 and endogenous VEGF expression in ovarian cells, which inhibited chemotherapy-induced GC apoptosis, promoted angiogenesis and regulated follicular development, thus partly reducing ovarian injury and improving ovarian function in rats with POI.

**Conclusions:**

hAD-MSC transplantation can improve ovarian function in rats with chemotherapy-induced POI at least partly through a paracrine mechanism. The presence of a paracrine mechanism accounting for hAD-MSC-mediated recovery of ovarian function might be attributed to the growth factors secreted by hAD-MSCs.

## Background

Premature ovarian insufficiency (POI) is a clinical syndrome characterized by oligomenorrhea or amenorrhea for at least 4 months, elevated follicle-stimulating hormone levels (FSH, > 25 mIU/ml on two occasions > 4 weeks apart) and low estradiol (E2) levels in women before the age of 40 years [1–3]. Untreated POI can induce multiple health risks, including menopausal symptoms, depression, anxiety, osteoporosis, increased risk of fracture and cardiovascular disease, and a decline in cognition [1]. POI affects approximately 1% and 0.1% of women before the ages of 40 and 30 years [2], respectively, and approximately 25% of POI is caused by iatrogenic factors, including chemotherapy [4]. It has been estimated and reported that there are approximately 1,000,000 new cancer diagnoses annually in adolescents and young adults (AYAs) aged 15 to 39 years worldwide [5]. AYA cancer survivors are increasingly overcoming cancer, resulting in a growing and substantial population of female cancer survivors in the reproductive age range [6]. Chemotherapy is a common method used to treat various malignancies and could induce ovarian failure and reduce fertility in young female patients [7]. Approximately 30% of women before the age of 35 years and 50% of women between the ages of 35 and 40 years who received chemotherapy were diagnosed with POI [8]. However, there is currently no effective therapy for POI. Therefore, it is important to examine and/or improve the treatment strategies for POI.

**Fig. 1:**
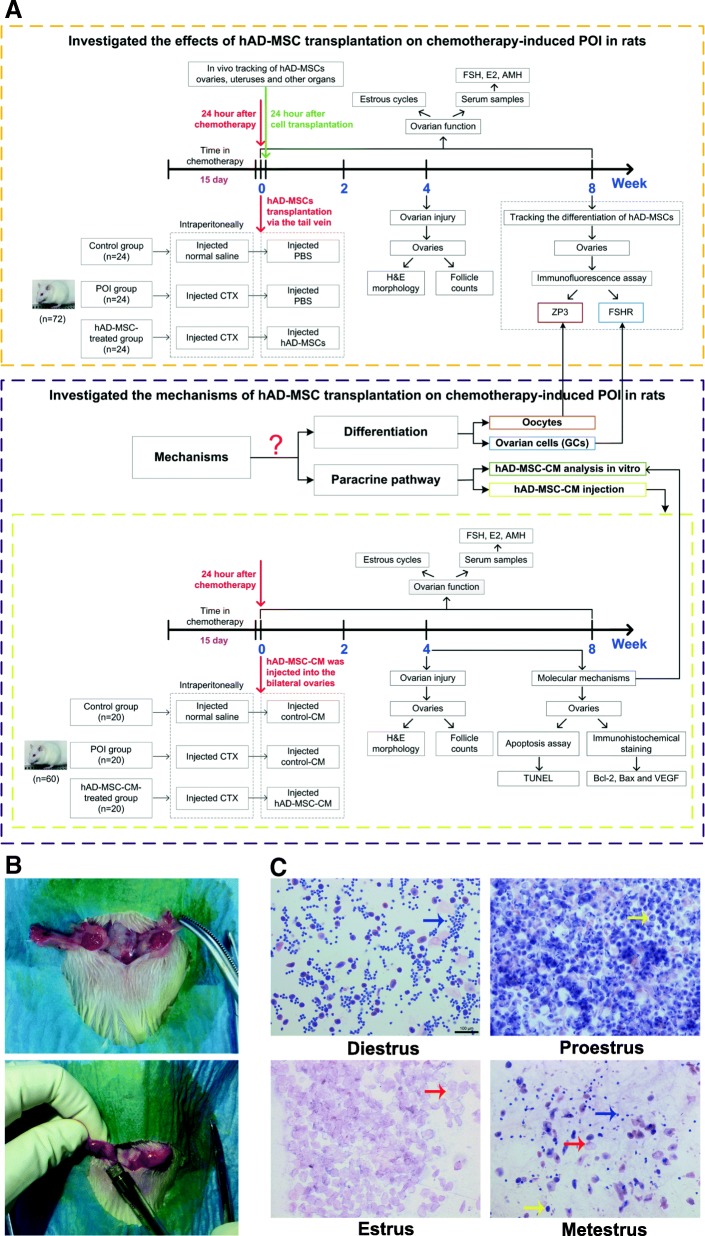
Experimental protocols and schematic. **a** A schematic of the experimental procedure used to explore the effects and mechanisms of hAD-MSC transplantation on chemotherapy-induced POI in rats. **b** Injection of CM into ovaries of SD rats. **c** The estrous cycle of SD rats (× 100). The yellow arrows indicate nucleated epithelial cells, the red arrows indicate cornified epithelium and the blue arrows indicate leukocytes. Scale bars, 100 μm

Regenerative medicine studies have shown that mesenchymal stem cell (MSC) transplantation may repair ovarian injury and improve ovarian function in animal models of POI, which provides an effective treatment method [9–11]. MSCs have multipotency, self-renewal capacity, low immunogenicity, immunomodulatory function, and injury chemotaxis [12–14]. Human amnion-derived mesenchymal stem cells (hAD-MSCs) have been demonstrated to not only have the features of MSCs [15, 16] but also have some phenotypic characteristics that are the same as embryonic stem cells [16, 17]. Moreover, the procedure used to obtain hAD-MSCs is safe, noninvasive, and ethical [18, 19]. These advantages make hAD-MSCs a promising *seed cell* for regenerative medicine and tissue engineering. Therefore, we investigated the effects of hAD-MSC transplantation on chemotherapy-induced POI in rats in this study (Fig. 1a).

Some studies have demonstrated the efficacy of stem cell transplantation on POI in animal models, and the mechanisms mainly consist of three elements [9, 20–22]. First, transplanted stem cells can differentiate into oocytes. Second, transplanted stem cells can differentiate into ovarian cells, which mainly include granulosa cells (GCs). Third, transplanted stem cells can restore ovarian function through the paracrine pathway. However, the mechanisms of hAD-MSC transplantation on POI remain unknown. Therefore, we investigated the mechanisms of hAD-MSC transplantation on chemotherapy-induced POI in rats in this study (Fig. 1a).

Several studies have suggested that the efficacy of MSC transplantation on POI is mainly attributed to the paracrine mechanism [9, 10]. MSCs can secrete a variety of paracrine/autocrine factors, including growth factors, chemokines, and colony-stimulating factors, which are called secretomes, that mediate diverse functions [23–25]. Some studies have shown that the MSC secretome could be therapeutic for the treatment of diseases [26–28]. Various paracrine factors secreted by MSCs can act directly, triggering intracellular mechanisms of injured cells, or act indirectly, promoting the secretion of functional active mediators in neighboring cells, which may attenuate tissue damage, inhibit apoptosis and fibrosis, promote angiogenesis, and modulate immune responses [25, 29]. MSC-conditioned media (CM) contains various factors and microvesicles secreted by MSCs that could be applicable in regenerative medicine [29]. There is evidence showing that stem cell transplantation can improve the local microenvironment in injured tissue by secreting various paracrine factors that can be harvested in CM, which are advantageous for repair and/or rejuvenation of injured cells and tissues [30, 31]. In this study, we further evaluated whether the mechanism of hAD-MSC transplantation on POI is through the paracrine pathway and whether CM containing various factors secreted by hAD-MSCs are efficient in treating rats with POI. To evaluate these mechanisms, we first analyzed the location and differentiation of transplanted hAD-MSCs in POI ovaries, and then the effects of hAD-MSC-CM on chemotherapy-induced POI in rats were investigated (Fig. 1a). These findings could provide new potential therapeutic strategies for patients with POI and evidence for the mechanisms of hAD-MSC transplantation on chemotherapy-induced POI.

## Methods

The experimental protocols were in compliance with the Helsinki Declaration and approved by the Ethics Committee of the Second Affiliated Hospital of Chongqing Medical University. All animal surgeries were performed under sodium pentobarbital anesthesia.

### Reagents

Cell Counting Kit-8 (CCK-8), penicillin, streptomycin, and a BCA protein assay kit were purchased from the Beyotime Institute of Biotechnology (Haimen, Jiangsu, China). Low-glucose Dulbecco’s modified Eagle’s medium (L-DMEM) and fetal bovine serum (FBS) were purchased from Gibco (Grand Island, NY, USA). PKH26 Red Fluorescent Cell Linker Kits were purchased from Sigma-Aldrich (St. Louis, MO, USA). 2-(4-Amidinophenyl)-6-indolecarbamidine dihydrochloride (DAPI) was purchased from Boster Biological Technology Co., Ltd. (Wuhan, Hubei, China). Cyclophosphamide (CTX) was purchased from Hengrui Medicine Co., Ltd. (Lianyungang, Jiangsu, China). Enzyme-linked immunosorbent assay (ELISA) kits for fibroblast growth factor 2 (FGF2), vascular endothelial growth factor (VEGF), insulin-like growth factor-1 (IGF-1), hepatocyte growth factor (HGF), and granulocyte-colony-stimulating factor (G-CSF) for humans and anti-Müllerian hormone (AMH), E2, and FSH for rats were purchased from Uscn Life Science (Wuhan, Hubei, China). A TUNEL apoptosis assay kit was purchased from Roche Applied Science (Basel, Switzerland). Rabbit anti-rat Bcl-2, Bax, and VEGF polyclonal antibodies were purchased from Proteintech Group, Inc. (Rosemont, IL, USA). Mouse anti-human ZP3 and rabbit anti-human follicle-stimulating hormone receptor (FSHR) monoclonal antibodies were purchased from Novus Biologicals (Littleton, CO, USA).

### Preparation of hAD-MSCs

Human term placentas from healthy donors (*n* = 30; clinically normal pregnancies) were collected from patients who received cesarean sections at the Second Affiliated Hospital of Chongqing Medical University, China. Amnions were separated from term placentas. Primary hAD-MSCs were isolated from term amnions. hAD-MSCs were isolated, cultured and identified according to our previous protocols, which have been published [15]. hAD-MSCs were cultured in L-DMEM supplemented with 100 U/mL penicillin, 0.1 mg/mL streptomycin, and 12% FBS. The third passage of hAD-MSCs was used for the subsequent experiments. hAD-MSCs were labeled with PKH26 Red Fluorescent Cell Linker Kits before transplantation [31, 32]. PKH26 expression in hAD-MSCs was verified using a fluorescence microscope (Nikon Corporation, Tokyo, Japan), and the cell labeling rate was detected by flow cytometry (BD Biosciences, San Diego, CA, USA). The growth curves of PKH26-labeled and unlabeled hAD-MSCs were investigated using a CCK-8 assay according to the manufacturer’s instructions.

### Preparation and detection of CM

When hAD-MSCs reached 60~70% confluency, the culture medium was replaced with fresh L-DMEM containing 0.5% FBS, and then hAD-MSCs were cultured for 5 days. The CM was collected after culture (approximately 5 × 10^5^ hAD-MSCs were cultured in 1 ml medium and considered as a unit), which was named hAD-MSC-CM. Fresh L-DMEM containing 0.5% FBS was used as control-CM. The concentration and molecular weight distribution of total proteins per unit in hAD-MSC-CM and control-CM were analyzed using a BCA protein assay kit and SDS-PAGE, respectively. Next, the hAD-MSC-CM and control-CM were concentrated 10 times using ultrafiltration centrifuge filter devices with a 3-kDa molecular weight cut-off value (Millipore, Billerica, MA, USA) and then filtered through 0.22 μm centrifugal filters (Millipore, Billerica, MA, USA) for sterilization. The concentrated CM was stored at − 80 °C until use in the following experiments.

### Animal protocols

Female Sprague-Dawley (SD) rats aged 10~12 weeks were purchased from the Experimental Animal Center of Chongqing Medical University.

To investigate the effects of hAD-MSC transplantation on chemotherapy-induced POI in rats, 72 female SD rats were randomly divided into three groups as follows: control group, POI group, and hAD-MSC-treated group (*n* = 24 in each group). The rats in the POI and hAD-MSC-treated groups were intraperitoneally injected with CTX (50 mg/kg/day on the first day and 8 mg/kg/day for the following 14 consecutive days) resuspended in normal saline for 15 days to establish the POI model [20], while the rats in the control group were intraperitoneally injected with an equivalent volume of normal saline. Then, the rats in the hAD-MSC-treated group were injected with 0.6 ml of cell suspension containing 4 × 10^6^ PKH26-labeled hAD-MSCs via the tail vein 24 h after chemotherapy, while the rats in the control and POI groups were injected with 0.6 ml of PBS. At 24 h and 2, 4, and 8 weeks after cell transplantation, six rats in each group were sacrificed under sodium pentobarbital anesthesia at the indicated time point, and samples were collected for the subsequent experiments (Fig. 1a).

To examine whether the mechanism of hAD-MSC transplantation on POI is through the paracrine pathway, the effects of hAD-MSC-CM on chemotherapy-induced POI in rats were investigated. Sixty female SD rats were randomly divided into three groups as follows: control group, POI group, and hAD-MSC-CM-treated group (*n* = 20 in each group). The rats in the POI and hAD-MSC-CM-treated groups were first injected with CTX to establish the POI model, as described above. At 24 h after chemotherapy, the rats in the three groups were anesthetized with sodium pentobarbital, and a 2~3-cm longitudinal incision was made 1.5 cm below the costovertebral angle on the dorsal midline of each rat to expose the bilateral ovaries (Fig. 1b). Then, 100 μL of concentrated hAD-MSC-CM was injected into the bilateral ovaries (50 μL in each ovary) of rats using a microliter syringe (volume 100 μL, Hamilton, Bonaduz, Switzerland) in the hAD-MSC-CM-treated group, while 100 μL of concentrated control-CM was injected into the bilateral ovaries of rats in the control and POI groups (Fig. 1b). At 2, 4, and 8 weeks after CM injection, six rats in each group were sacrificed under sodium pentobarbital anesthesia at the indicated time point, and samples were collected for the subsequent experiments (Fig. 1a).

Vaginal smears from rats were obtained at 9:00 am daily to observe the estrous cycle in each group. The regular estrous cycle of rats consists of four sequential stages as follows: proestrus, estrus, metestrus, and diestrus, which were identified according to the presence or absence of cornified epithelium, nucleated epithelial cells, and leukocytes [21] (Fig. 1c). Rats that went through at least two consecutive regular estrous cycles were included in the experiments. The length of an estrous cycle was determined as the number of days between two non-consecutive days during which estrus cytology was observed [25]. The average length of regular estrous cycles in the rats was 4.4 days (*n* = 18), ranging from 4 to 5 days. Irregular estrous cycles were defined as follows [25, 33, 34]: (i) a prolonged estrous cycle, the rat has estrous cycle, but irregular, and the length of estrous cycle is more than 5 days; and (ii) irregular duration of diestrus and proestrus, the estrous cycle of rat disappears, and the rat remains in persistent diestrus or in persistent diestrus and proestrus until the end of the observation period.

### Tracking of transplanted PKH26-labeled hAD-MSCs

PKH26-labeled hAD-MSCs were injected into rats with POI via the tail vein. To track the distribution of the transplanted hAD-MSCs in rats, the ovaries, uteruses, livers, hearts, lungs, spleens, kidneys, and brains were processed into frozen sections at 24 h after hAD-MSC transplantation. The sections were fixed, washed, and incubated with DAPI. Then, all sections were imaged using a fluorescence microscope (Nikon Corporation, Tokyo, Japan).

### Ovarian morphology analysis and follicle counts

To analyze the ovarian morphology and follicle counts, ten ovaries from each group were collected at 4 weeks after hAD-MSC transplantation or CM injection. Ovaries were fixed with 4% paraformaldehyde, dehydrated, paraffin embedded, and cut into 5 μm sections. The sections were stained with hematoxylin and eosin (HE). The ovarian morphology was observed using an optical microscope (Olympus Corporation, Tokyo, Japan), and the number of follicles was counted, as described previously [9, 35]. For follicle counting, the follicles were classified as atretic, primordial, primary, secondary, and preovulatory follicles [22, 23].

### Apoptosis assay

To investigate the effects of hAD-MSC-CM on ovarian cell apoptosis induced by CTX in POI rats, a TUNEL apoptosis assay kit was used to detect ovarian cell apoptosis in each group at 4 weeks after CM injection according to the manufacturer’s instructions. Nuclei of apoptotic cells were stained dark brown. Sections were observed and imaged using an optical microscope (Olympus Corporation, Tokyo, Japan). The sections were evaluated using a double-blind method and analyzed by two pathologists.

### Immunohistochemical staining

The expression of Bax, Bcl-2, and VEGF in the ovaries of the control, POI, and hAD-MSC-CM-treated groups was analyzed using immunohistochemical staining at 4 weeks after CM injection. Ovarian tissue sections were obtained and incubated with 5% bovine serum albumin (BSA) for 2 h. The sections were incubated with specific primary antibodies for Bax, Bcl-2, and VEGF overnight at 4 °C. After washing, the sections were incubated with the corresponding secondary antibodies followed by horseradish peroxidase. Then, the sections were washed, stained with 3,3′-diaminobenzidine (DAB), and counterstained using hematoxylin. The sections were observed and imaged using an optical microscope (Olympus Corporation, Tokyo, Japan). The sections were evaluated using a double-blind method. The sections were analyzed by two pathologists using a semiquantitative scoring system, as previously described [24, 25], according to the staining intensity graded as 3 (brown), 2 (light brown), 1 (light yellow), or 0 (no color), and the number of positive cells graded as 4 (> 75%), 3 (51~75%), 2 (25~50%), 1 (5~25%), or 0 (< 5%). The total score was the sum of the two grades, which was named the immunoreactivity score (MVIS). Ten high-power fields (HPFs, × 400) were randomly chosen from five sections in each group for scoring. The median value of the MVIS for each group was calculated.

### ELISA

The levels of cytokines, i.e., FGF2, VEGF, IGF-1, HGF, and G-CSF, secreted by hAD-MSCs were analyzed using ELISA kits. hAD-MSCs were seeded at a density of 1 × 10^5^ cells/mL onto a 6-well plate. When the hAD-MSCs reached 60~70% confluency, the culture medium was replaced with serum-free medium. After 72 h, the cell supernatant was collected to analyze the cytokines according to the manufacturer’s instructions.

Serum levels of AMH, E2, and FSH at the indicated time points (0, 2, 4, and 8 weeks after cell transplantation or CM injection) were analyzed using ELISA kits according to the manufacturer’s instructions.

### Immunofluorescence assay

The differentiation of hAD-MSCs in the ovaries of the hAD-MSC-treated group was analyzed using immunofluorescent staining at 8 weeks after cell transplantation. The ovaries were fixed with optimal cutting temperature compound (Sakura Finetek USA, Inc., Torrance, CA, USA) and cut into fresh 10 μm sections. The sections were fixed, washed, and incubated with 5% BSA for 2 h. Then, the sections were incubated with specific primary antibodies for human ZP3 and FSHR overnight at 4 °C. After washing, the sections were incubated with corresponding secondary antibodies conjugated with DyLight488. The sections were counterstained with DAPI and imaged using a laser scanning confocal microscope (Nikon Corporation, Tokyo, Japan).

### Statistical analysis

Statistical analysis was conducted using SPSS 22.0 software (IBM, NY, USA). Data with normal distribution are presented as the mean ± standard deviation (SD), and one-way analysis of variance (ANOVA) and independent samples *t* test were used for multiple-group and two-group comparisons, respectively. Data with skewed distributions are presented as medians and quartile ranges, and nonparametric Kruskal-Wallis tests and Wilcoxon rank tests were used for multiple-group and two-group comparisons, respectively. Statistical significance was set at *P* < 0.05.

## Results

### Characterization of hAD-MSCs

**Fig. 2:**
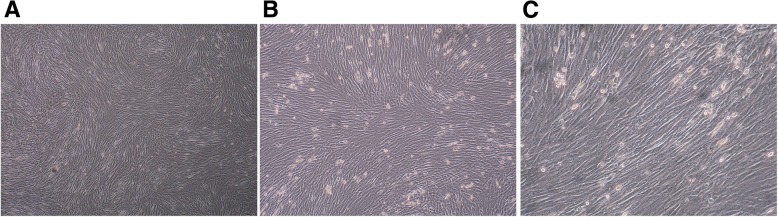
Morphology of hAD-MSCs (**a** × 40, **b** × 100, **c** × 200)

In our experiments, isolated cells on the third passage showed a fibroblastic morphology (Fig. 2). These isolated cells were identified as hAD-MSCs by our group in previous studies [15, 35], which showed the common characteristics of multipotent MSCs.

### In vivo tracking of hAD-MSCs

**Fig. 3:**
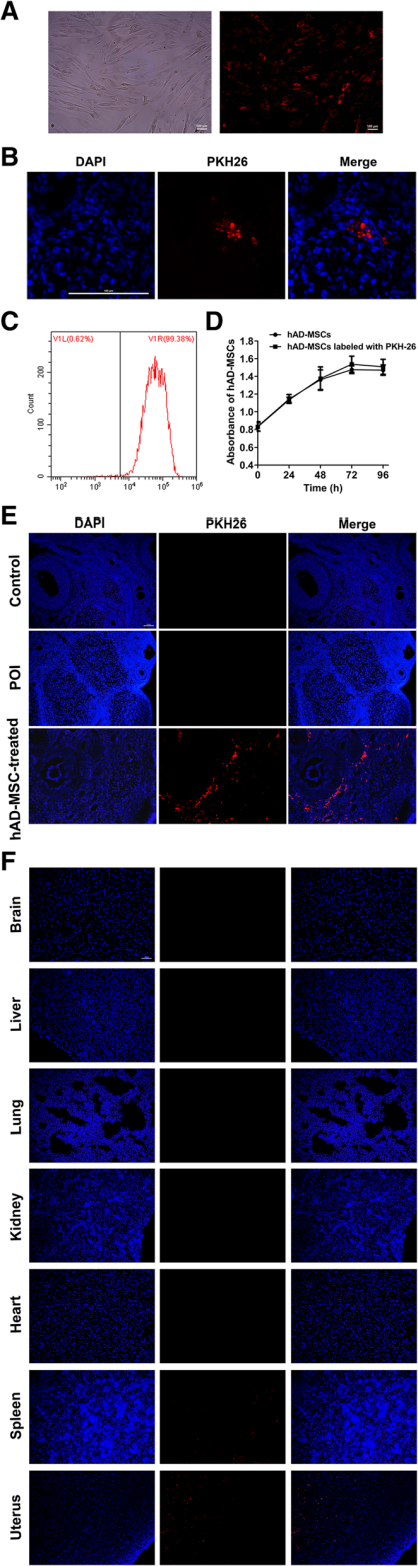
Tracking of hAD-MSCs in vivo. **a**, **b** PKH26-labeled hAD-MSCs showed red fluorescence in vitro (**a** × 100) and in vivo (**b** × 800). **c** The labeling rate of PKH26-labeled hAD-MSCs was analyzed using flow cytometry. **d** The growth curves of PKH26-labeled and unlabeled hAD-MSCs were analyzed using a CCK-8 assay (*n* = 6). **e**, **f** Transplanted PKH26-labeled hAD-MSCs were observed at 24 h after cell transplantation in ovaries (**e** × 100) and other organs (**f** × 100) of SD rats in the hAD-MSC-treated group. *P < 0.05 and **P < 0.01. Scale bars, 100 μm

To track and locate the position of transplanted hAD-MSCs in vivo, hAD-MSCs were prelabeled with PKH26. PKH26-labeled hAD-MSCs showed red fluorescence (Fig. 3a, b). The cell labeling rate of PKH26-labeled hAD-MSCs was 99.21 ± 0.33% (Fig. 3c). There was no significant difference in cell proliferation and activity between PKH26-labeled and unlabeled hAD-MSCs (Fig. 3d).

The location of transplanted PKH26-labeled hAD-MSCs in rats was traced at 24 h after cell transplantation in the hAD-MSC-treated group. The results showed that the transplanted cells mainly homed to ovaries (Fig. 3e), which was the primary target organ injured by CTX [26, 27]. Some red-dotted fluorescent signals were observed in uteruses and spleens (Fig. 3f). However, a red fluorescent signal was not observed in the livers, hearts, lungs, kidneys, and brains (Fig. 3f).

### Changes in ovarian function and histology after hAD-MSC transplantation

**Fig. 4:**
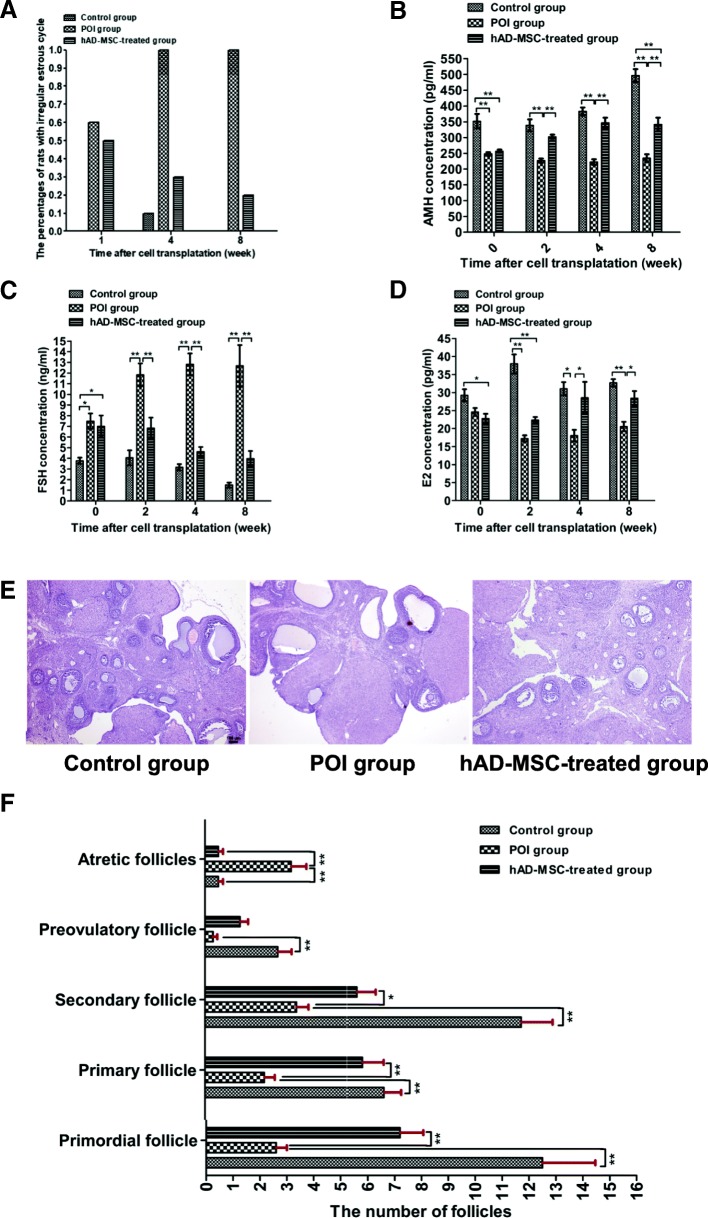
Changes in ovarian function and histology after hAD-MSC transplantation. **a** The percentage of rats with abnormal estrous cycles was observed at 1, 4, and 8 weeks after hAD-MSC transplantation. **b**–**d** Serum levels of AMH (**b**), FSH (**c**), and E2 (**d**) were evaluated at 0, 2, 4, and 8 weeks after hAD-MSC transplantation. **e**, **f** Changes in the histology of ovaries were analyzed using HE staining (**e** × 40), and the number of follicles at different stages was counted and compared (**f**) in the three groups at 4 weeks after hAD-MSC transplantation. *P < 0.05 and **P < 0.01. Scale bars, 100 μm

To investigate the effects of hAD-MSC transplantation on ovarian function in rats with POI, estrous cycles, and serum levels of FSH, E2, and AMH were analyzed. In the control group, 100% of the rats had regular estrous cycles (Fig. 4a). In the POI group, 60% of the rats had irregular estrous cycles, including a prolonged estrous cycle and irregular duration of diestrus and proestrus, in the first week after chemotherapy, and 100% of rats had irregular estrous cycles from the fourth week after chemotherapy. Compared to the POI group, the percentages of rats with irregular estrous cycles were lower in the hAD-MSC-treated group from the first week after hAD-MSC transplantation (Fig. 4a).

Compared to the control group, the AMH levels were significantly lower in the POI and hAD-MSC-treated groups after chemotherapy (*P* < 0.01; Fig. 4b), while the FSH levels were significantly higher (*P* < 0.05; Fig. 4c). Compared to the POI group, the AMH level was significantly higher in the hAD-MSC-treated group, starting from the second week after hAD-MSC transplantation (*P* < 0.01; Fig. 4b), while the FSH level was significantly lower (*P* < 0.01; Fig. 4c). Moreover, compared to the control group, the E2 levels were lower in the POI and hAD-MSC-treated groups after chemotherapy (Fig. 4d). Compared to the POI group, the E2 level was significantly higher in the hAD-MSC-treated group starting from the fourth week after hAD-MSC transplantation (*P* < 0.05; Fig. 4d). These results demonstrated that hAD-MSC transplantation improved ovarian function in rats with POI.

Ovaries in the control, POI and hAD-MSC-treated groups were collected for histological analysis at 4 weeks after hAD-MSC transplantation. Compared to the control group, the number of preovulatory, secondary, primary and primordial follicles was significantly lower in the POI group (*P* < 0.01; Fig. 4e and f), while the number of atretic follicles was significantly greater (*P* < 0.01; Fig. 4e, f). Furthermore, compared to the POI group, the number of secondary, primary, and primordial follicles was significantly greater in the hAD-MSC-treated group (*P* < 0.05; Fig. 4e, f), while the number of atretic follicles was significantly lower (*P* < 0.01; Fig. 4e, f). These results demonstrated that hAD-MSC transplantation reduced ovarian injury in rats with POI.

### Tracking the differentiation of hAD-MSCs in ovaries

**Fig. 5:**
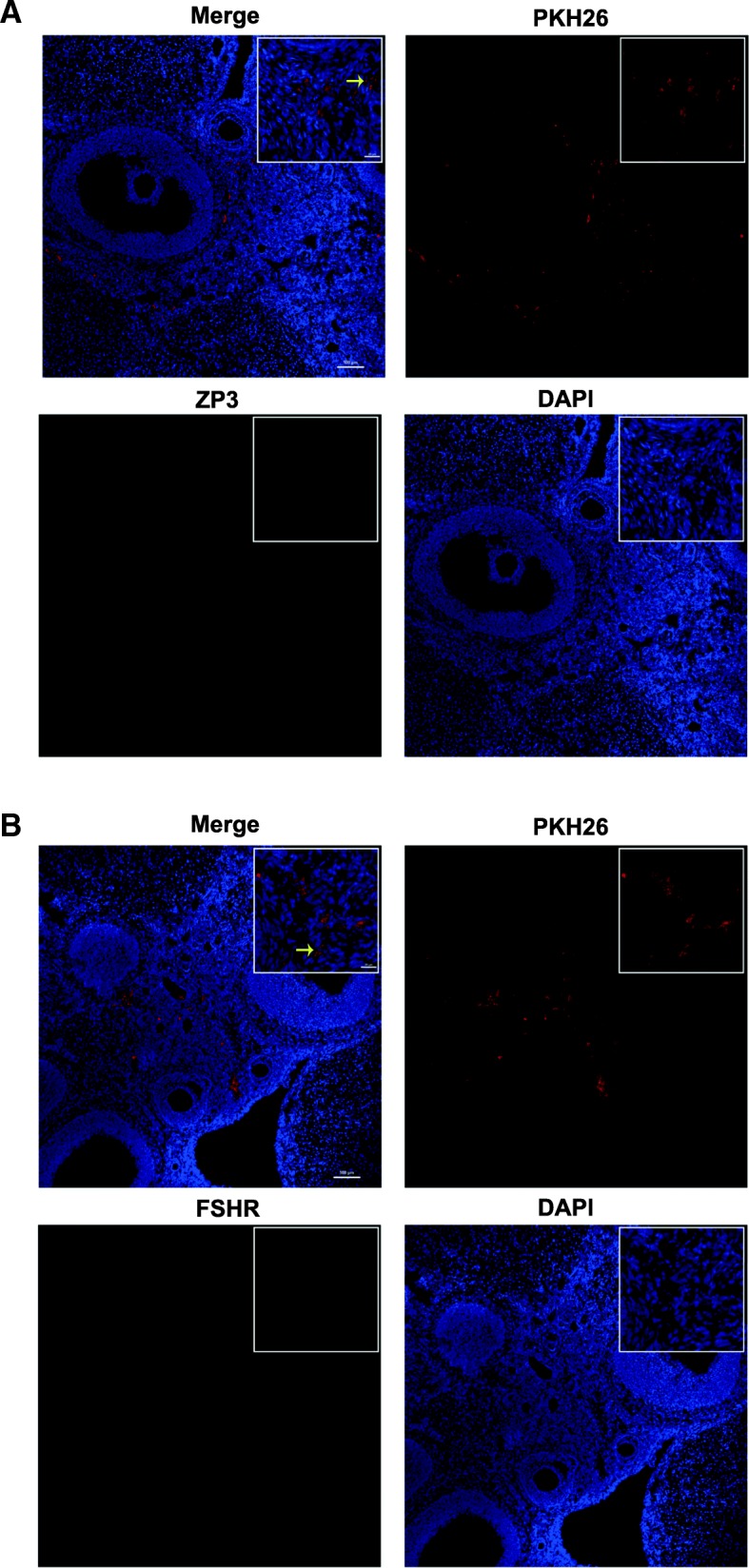
Tracking the differentiation of hAD-MSCs in ovaries. The expression of ZP3 (**a** × 100 and × 800) and FSHR (**b** × 100 and × 800) in hAD-MSCs was observed at 8 weeks after hAD-MSC transplantation in ovaries. The yellow arrows indicate transplanted hAD-MSCs. Scale bars, 100 μm; Scale bars, 20 μm

To investigate the mechanisms of hAD-MSC transplantation on chemotherapy-induced POI, the differentiation of transplanted hAD-MSCs in ovaries was analyzed. The results showed that PKH26-labeled hAD-MSCs were only located in the interstitium of ovaries, rather than in follicles, after transplantation in the hAD-MSC-treated group (Figs. 3e and 5a, b). Furthermore, green fluorescent signals were not observed in ovaries at 8 weeks after hAD-MSC transplantation in the hAD-MSC-treated group (Fig. 5a, b), which indicated that the transplanted hAD-MSCs did not express ZP3 and FSHR, the typical markers of oocytes and GCs, respectively. These results demonstrated that the transplanted hAD-MSCs did not differentiate into oocytes and GCs in POI ovaries.

### Analysis of CM derived from hAD-MSCs

**Fig. 6:**
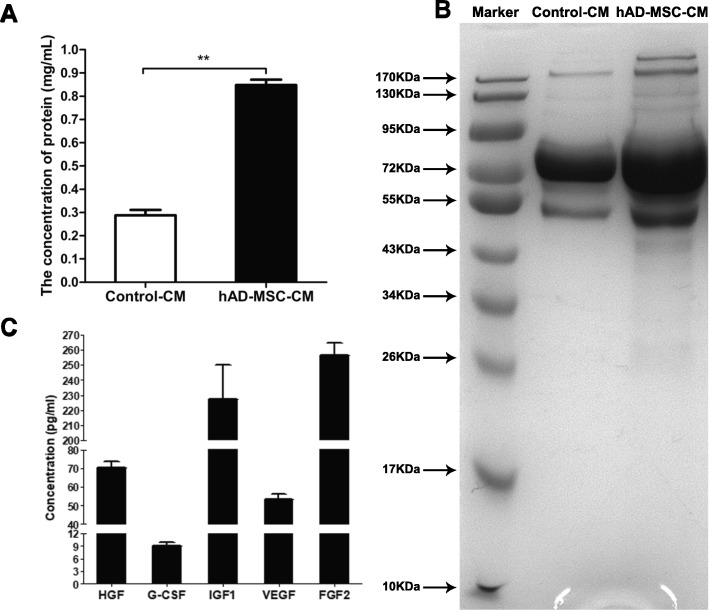
Analysis of CM derived from hAD-MSCs. The protein concentration (**a**) and molecular weight distribution of proteins (**b**) and cytokines secreted by hAD-MSCs (**c**) in CM were analyzed. *P < 0.05 and **P < 0.01

To better understand the paracrine components derived from hAD-MSCs in CM, the protein concentration and molecular weight distribution of proteins and cytokines secreted by hAD-MSCs in CM were analyzed. The results showed that the protein concentration in the hAD-MSC-CM was significantly greater than that in the control-CM (*P* < 0.01; Fig. 6a). The molecular weight distribution of proteins in the hAD-MSC-CM ranged from 20 to 200 kDa, but mainly ranged from 43 to 95 kDa, which was different from that in the control-CM (Fig. 6b).

The levels of cytokines secreted by hAD-MSCs were analyzed, including G-CSF, FGF2, IGF-1, HGF, and VEGF, which likely play important roles in repairing ovarian injuries and restoring ovarian functions in animals with POI using stem cells [28, 29, 36]. The results showed that hAD-MSCs secreted G-CSF, FGF2, IGF-1, HGF, and VEGF and especially expressed high levels of FGF2, IGF-1, HGF, and VEGF (Fig. 6c).

### Changes in ovarian function and histology after hAD-MSC-CM injection

**Fig. 7:**
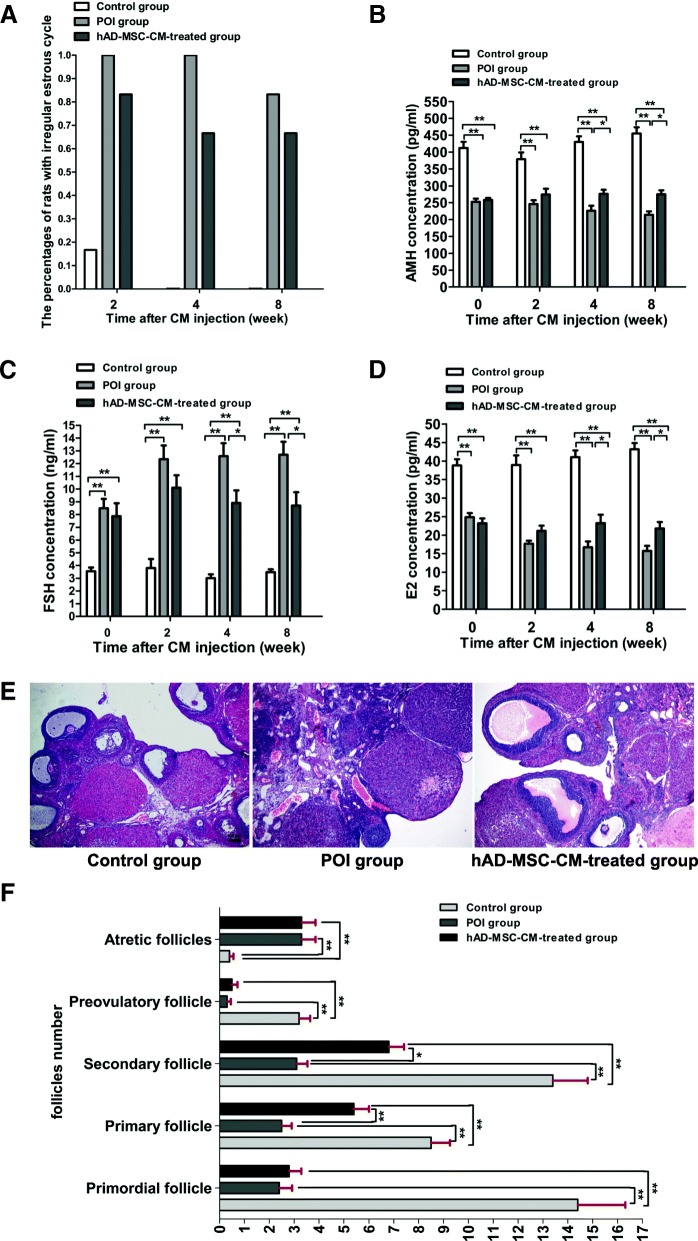
Changes in ovarian function and histology after hAD-MSC-CM injection. **a** The percentage of rats with abnormal estrous cycles was observed at 2, 4, and 8 weeks after CM injection. **b**–**d** Serum levels of AMH (**b**), FSH (**c**), and E2 (**d**) were evaluated at 0, 2, 4, and 8 weeks after CM injection. **e**, **f** Changes in the histology of ovaries were analyzed using HE staining (**e** × 40), and the number of follicles at different stages was counted and compared (**f**) in the three groups at 4 weeks after CM injection. *P < 0.05 and **P < 0.01. Scale bars, 100 μm

To further examine whether the mechanism of hAD-MSC transplantation on POI is through the paracrine pathway, the effects of hAD-MSC-CM on chemotherapy-induced POI in rats were investigated. The results showed that compared to the POI group, the percentages of rats with irregular estrous cycles were lower in the hAD-MSC-CM-treated group from the second week after hAD-MSC-CM injection (Fig. 7a), and the levels of AMH and E2 were significantly greater in the hAD-MSC-CM-treated group starting from the fourth week after hAD-MSC-CM injection (*P* < 0.05; Fig. 7b, d), while the FSH level was significantly lower (*P* < 0.05; Fig. 7c). These results demonstrated that hAD-MSC-CM injection partially improved ovarian function in rats with POI.

Furthermore, ovaries in the control, POI, and hAD-MSC-CM-treated groups were also collected for histological analysis at 4 weeks after hAD-MSC-CM injection. Compared to the POI group, the number of primary and secondary follicles was significantly greater in the hAD-MSC-CM-treated group (*P* < 0.05; Fig. 7e, f). The results demonstrated that the hAD-MSC-CM injection partially reduced ovarian injury in rats with POI.

### Effects of hAD-MSC-CM on chemotherapy-induced ovarian GC apoptosis in POI rats

**Fig. 8:**
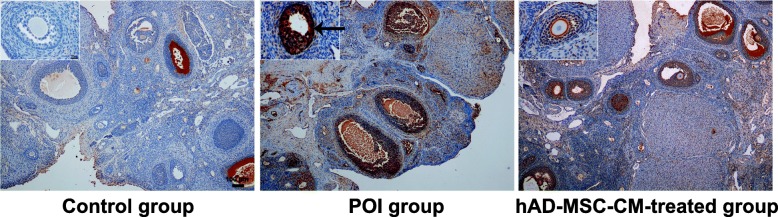
The effects of hAD-MSC-CM on chemotherapy-induced ovarian GC apoptosis in POI rats. Representative images of the TUNEL apoptosis assay for ovaries from the three groups are shown (× 40 and × 400). Dark brown cells representing ovarian apoptotic GCs are indicated by black arrows. Scale bars, 100 μm; Scale bars, 20 μm

To investigate the effects of hAD-MSC-CM on ovarian GC apoptosis induced by chemotherapy, TUNEL apoptosis staining was conducted 4 weeks after hAD-MSC-CM injection. The results showed that compared to the control group, the number of apoptotic GCs in ovaries was significantly greater in the POI group (Fig. 8). Furthermore, compared to the POI group, the number of apoptotic GCs in ovaries was significantly lower in the hAD-MSC-CM-treated group (Fig. 8). The results demonstrated that hAD-MSC-CM injection reduced chemotherapy-induced ovarian GC apoptosis in POI rats.

### Effects of hAD-MSC-CM on the expression of Bcl-2, Bax, and VEGF in the ovaries of POI rats

**Fig. 9:**
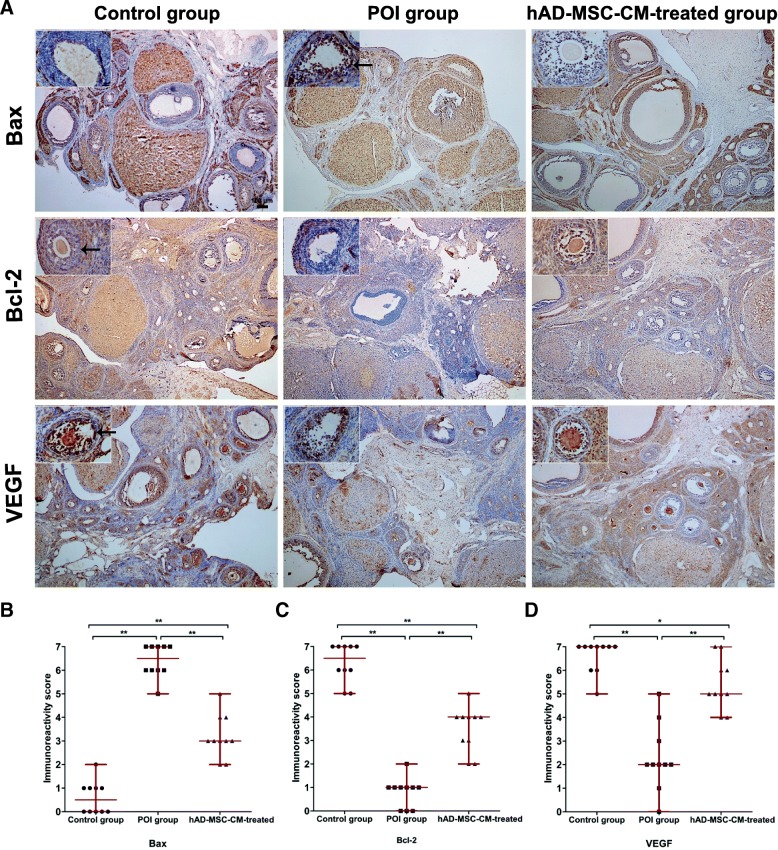
The effects of hAD-MSC-CM on the expression of Bcl-2, Bax, and VEGF in the ovaries of POI rats. Representative immunohistochemistry images (**a** × 40 and × 400) and semiquantitative analysis of Bax, Bcl-2, and VEGF expression (**b**–**d**) of ovaries from the three groups are shown (*n* = 10). Immunostained cells (brown cells) are indicated by black arrows. Each dot in graphs **b**, **c**, and **d** represents the value across ten HPFs randomly chosen from five sections in each group. The bars and error bars indicate the medians and ranges, respectively. *P < 0.05 and **P < 0.01. Scale bars, 100 μm; Scale bars, 20 μm

To investigate the likely molecular mechanisms of hAD-MSC-CM on improving ovarian function and reducing ovarian injury in rats with POI, the expression of two key apoptosis-related proteins, Bcl-2 and Bax, and one key angiogenic factor, VEGF, in the ovaries from the three groups were analyzed. The results showed that compared to the control group, the MVIS of Bax expression was significantly greater in the POI group (*P* < 0.01; Fig. 9a, b), while the MVIS of Bcl-2 expression was significantly lower (*P* < 0.01; Fig. 9a, c). Moreover, compared to the control group, the MVIS of VEGF expression was also significantly lower in the POI group (*P* < 0.01; Fig. 9a, d). Furthermore, compared to the POI group, the MVIS of Bax expression was significantly lower in the hAD-MSC-CM-treated group (*P* < 0.01; Fig. 9a, b), while the MVIS of Bcl-2 expression was significantly greater (*P* < 0.01; Fig. 9a, c). Moreover, compared to the POI group, the MVIS of VEGF expression was also significantly greater in the hAD-MSC-CM-treated group (*P* < 0.01; Fig. 9a, d). These results demonstrated that chemotherapy-induced POI could be associated with the decreased expression of Bcl-2 and VEGF and increased expression of Bax in ovaries, and hAD-MSC-CM promoted Bcl-2 and VEGF expression and inhibited Bax expression in the ovaries of POI rats.

## Discussion

Currently, there is no effective treatment strategy for chemotherapy-induced POI. Recently, regenerative medicine research has shown that MSC transplantation may provide an effective treatment method to restore ovarian function in animals with POI [9, 11]. In this study, CTX was used to establish the chemotherapy-induced POI rat model, according to a previously reported protocol [20, 35], which has been shown to cause POI in humans. Previous studies have shown that hAD-MSCs may be a promising *seed cell* for regenerative medicine and tissue engineering [16, 35]. In our study, after hAD-MSC transplantation, the changes of ovarian morphology and follicle counts, representing ovarian injury [30, 35], were analyzed in the rats of control, POI, and hAD-MSC-treated groups. On the other hand, the estrous cycles and serum levels of FSH, E2, and AMH, representing ovarian function [9, 35], were also tested in those rats. Our results demonstrated that hAD-MSC transplantation reduced ovarian injury and improved ovarian function in rats with chemotherapy-induced POI. As we know, follicular growth is chiefly regulated by gonadotropins. The gonadotropins FSH is produced by the anterior pituitary gonadotroph cells and responsible for ovarian follicular stimulation and growth. FSHR is mainly expressed on GCs in follicles [37]. E2 is produced by theca cells and GCs in follicles. Rising E2 level provides negative feedback on pituitary FSH secretion in the hypothalamus-pituitary-ovary axis. AMH can be secreted by the GCs of primary and secondary follicles and inhibits the initiation of primordial follicle growth [38]. As the ovarian primordial follicle count decreases, the serum AMH concentration also decreases, making this hormone an ideal candidate for the early detection of ovarian reserve depletion [38]. In this study, CTX was used to establish a POI model, which can induce GC apoptosis, follicular loss, vascular damage, and tissue fibrosis in ovaries and cause ovarian failure [26, 39]. Our results showed that CTX induced a decrease in the number of secondary, primary, and primordial follicles, GC apoptosis, and tissue fibrosis in the ovaries of rats in the POI group, and resulted in decreased serum levels of E2 and AMH. The decreased E2 level interrupted the negative feedback, and the serum FSH level increased. Although rising FSH level stimulated residual follicular growth in the POI ovaries, it accelerated follicle depletion and then failed to stimulate follicular growth due to GC apoptosis and follicle depletion, which further resulted in low E2 level. We found that hAD-MSC transplantation increased the number of secondary, primary, and primordial follicles and reduced ovarian injury in rats with POI, and resulted in increased serum levels of E2 and AMH in the hAD-MSC-treated group. Rising E2 level provided negative feedback on FSH secretion, and serum FSH level decreased in response to rising E2 level in the hAD-MSC-treated group. However, our results showed that, compared to the control group, the levels of E2, FSH, and AMH were not completely restored in the hAD-MSC-treated group, which may be related to the number of follicles at various stages and the structure of injured POI ovaries that were not completely restored by hAD-MSC transplantation in the hAD-MSC-treated group. In the future, hAD-MSC transplantation may provide a potential therapeutic strategy to reduce ovarian injury and improve ovarian function in female patients receiving chemotherapy. To improve the understanding of this potential therapeutic strategy, we further examined the underlying mechanisms.

Several studies have found that stem cells might repair ovarian tissue damage and improve ovarian function through the paracrine mechanism [23, 30]. In our study, we demonstrated that although hAD-MSC transplantation restored ovarian function in rats with POI, transplanted hAD-MSCs in the hAD-MSC-treated group were only located in the interstitium of ovaries, rather than in follicles, and did not express the typical markers of oocytes and GCs, which are ZP3 and FSHR, respectively. These results indicated that the transplanted hAD-MSCs did not differentiate into oocytes or GCs, and the transplanted hAD-MSCs may improve ovarian function through a paracrine mechanism in rats with chemotherapy-induced POI.

To confirm whether the mechanism of hAD-MSC transplantation on chemotherapy-induced POI is through the paracrine pathway, hAD-MSC-CM containing various cytokines and microvesicles secreted by hAD-MSCs was concentrated and injected into the bilateral ovaries of POI rats in this study. The results showed that hAD-MSC-CM injection partially reduced ovarian injury and improved ovarian function in rats with POI. Previously reported evidence has suggested that stem cells likely promote the recovery of injured tissue through secreted factors [40–42]. Previous studies have shown that the human amniotic membrane is a biological source of matrix that can secrete multiple growth factors, including VEGF, HGF, FGF2, transforming growth factor beta (TGF-ß), and keratinocyte growth factor (KGF) [43, 44]. Previous studies have also demonstrated that human MSCs can secrete various cytokines, including VEGF, FGF2, HGF, IGF-1, G-CSF, IL-6, IL-8, IL-10, IL-11, and IL-15 [29, 36]. However, whether hAD-MSCs can secrete those cytokines remains unknown. To investigate the specific molecular mechanisms of hAD-MSC-mediated repair of ovarian function, we analyzed the potential functional components in hAD-MSC-CM, with a focus on VEGF, FGF2, IGF-1, HGF, and G-CSF, which were more likely to play important roles in repairing ovarian injury and restoring ovarian function in animals with POI using stem cells [9, 28, 29]. Our results demonstrated that hAD-MSCs secreted those cytokines and especially expressed high levels of VEGF, FGF2, IGF-1, and HGF. Although follicular growth is chiefly regulated by gonadotropins in a variety of monovular and polyovular species [45, 46], the regulation of certain local growth factors, such as VEGF, FGF2, HGF, and IGF-1, is inevitable, without which follicles fail to grow further [47–49]. Gonadotropins also stimulate ovarian follicular angiogenesis partly by regulating the expression of those growth factors [47, 50], and angiogenesis is an important prerequisite for follicular development and maturation [51]. Additionally, those growth factors have been shown to stimulate cell proliferation and have antiapoptotic effects in GCs [48, 52], and GCs support oocyte survival and are required for follicular development [51]. VEGF is a key angiogenic factor that has been demonstrated to play an important role in the growth, recruitment, and establishment of dominance of antral follicles in buffalo, cattle, and humans [53, 54]. VEGF can also inhibit cell death and delay apoptosis [55]. Previous studies have demonstrated that VEGF can suppress GC apoptosis and inhibit follicular atresia resulting from GC apoptosis in the mammalian ovary [56]. Moreover, the initiation and further progression of follicular angiogenesis in the ovary is primarily regulated by VEGF [47, 50]. It has been reported that FGF2 can promote angiogenesis, vessel growth and survival of injured cells [32, 57]. FGF2 is able to behave in an autocrine manner on several cell functions required for angiogenesis, including proteinase production, integrin expression, and cell proliferation and migration [58]. Studies have shown that FGF2 may promote GC survival and steroidogenesis in an autocrine and paracrine manner [59]. FGF2 may be responsible for GC proliferation and the prevention of apoptosis and may play an important role in follicular development by affecting cell differentiation and ovulation processes [51]. IGF-1, a potent stimulator of cell proliferation and differentiation, regulates GC apoptosis and steroidogenesis during follicular development [60]. IGF-1 has been observed to promote cell proliferation and prevent apoptosis in cultured follicles in vitro [52]. It has been shown that IGF-1 not only stimulates VEGF as well as the production of progesterone by GCs but also synergizes with gonadotropins to maintain secretory activity in GCs in monkeys [60]. HGF, which can act as an anti-inflammatory or antiapoptotic signal in various organs, stimulates angiogenesis, organ morphogenesis, and cell proliferation and motility [51]. Numerous biological functions of HGF have been reviewed, including the promotion of tissue regeneration in organs after injuries [61]. HGF has also been demonstrated to be an angiogenic factor complementary to FGF2 and VEGF [61]. Previous studies have shown that HGF is an important paracrine/autocrine regulator of theca cell and GC growth and steroidogenesis in ovaries [62, 63]. HGF regulates numerous key functions in ovaries, including cell growth, steroidogenesis, and apoptosis of GCs and/or theca cells, which collectively regulate follicular growth and differentiation [48]. HGF has also been found to play a key role in follicular angiogenesis [51]. Based on these findings, previous studies have shown that growth factors in ovaries can facilitate folliculogenesis, follicular growth, and steroidogenesis, increase the number of follicles, reduce the onset of apoptosis in GCs and follicles, and promote angiogenesis in injured ovaries [47, 48, 52, 64, 65]. In our study, CTX was used to establish a POI model, which can induce GC apoptosis, follicular loss, vascular damage, and tissue fibrosis in ovaries and cause ovarian failure [26, 39]. FGF2, IGF-1, HGF, and VEGF secreted by hAD-MSCs were analyzed in hAD-MSC-CM. Our results showed that hAD-MSC-CM injection inhibited chemotherapy-induced ovarian GC apoptosis, increased the number of primary and secondary follicles and promoted local VEGF expression in the ovaries of POI rats, thus reducing ovarian injury and improving ovarian function in rats with POI. Therefore, it is plausible that these growth factors might play important roles in hAD-MSC-mediated recovery of ovarian function in rats with POI by inhibiting apoptosis and promoting angiogenesis and follicular growth in ovaries. Although the growth factors secreted by hAD-MSCs might play important roles in this process, the molecular mechanisms of hAD-MSC-mediated repair of ovarian function after chemotherapy are complicated, and further detailed studies are needed.

Furthermore, our results from this study also showed that compared to hAD-MSC-CM injection, hAD-MSC transplantation was more efficient in reducing ovarian injury and improving ovarian function in rats with POI. We speculate that there are two main possible reasons for these results. One reason is that the discrepancy between the culture medium in vitro and the chemotherapy-induced pathological environment of the ovary in vivo might have caused differences in the secretory function and viability of hAD-MSCs. The second reason is that the concentration and dose of hAD-MSC-CM used in this study may not have been optimal. Therefore, in the subsequent experiments, we will test a variety of concentrations and doses of hAD-MSC-CM for the treatment of POI rats. Nevertheless, from the results obtained in this study, we demonstrated that hAD-MSC transplantation improved ovarian function in rats with POI at least partly through a paracrine mechanism.

In this study, we demonstrated that chemotherapy-induced POI may be associated with the decreased expression of Bcl-2 and VEGF and increased expression of Bax in ovaries, and hAD-MSC-CM can promote Bcl-2 and VEGF expression and inhibit Bax expression in the ovaries of POI rats. The maintenance of cell survival in normal tissues depends on extracellular viability factors, including growth factors, extracellular matrix molecules and serum, which transmit signals into the interior of cells, leading to the expression of molecules that promote cell viability [55]. Therefore, the injection of hAD-MSC-CM containing various growth factors into the ovaries promoted ovarian cell viability. Several intracellular molecules are involved in maintaining cell viability, and the Bcl-2 family seems to play a prominent and central role in this respect [66]. Bcl-2 is an intracellular protein that inhibits apoptosis and prolongs cell survival [67]. Bax is a Bcl-2 homolog, and overexpression of Bax accelerates apoptosis [68]. Previous studies have demonstrated that Bcl-2 family members play important roles in regulating ovarian cell apoptosis and determining the fate of ovarian GCs and follicles [35, 69]. FGF2 can upregulate Bcl-2 expression, inhibit cell death, and delay apoptosis [55]. Previous studies have shown that FGF2 may initially protect cells independent of Bcl-2 and may subsequently upregulate Bcl-2 [55]. Primary culture of GCs treated with FGF2 at different doses has revealed that the mRNA and protein expression of Bax in GCs decreased after FGF2 incubation in a dose- and time-dependent manner, which suggests the cytoprotective role of FGF2 on GCs [59]. VEGF has an important role in modulating follicular growth, dominance, and steroidogenesis in ovaries [70]. Mishra et al. [71] found that the relative abundance of Bax mRNA decreased after 72 h of VEGF incubation in cultured GCs. Abramovich et al. [72] found that the local inhibition of VEGF activity in the ovary significantly increased the protein expression levels of Bax and decreased those of Bcl-2, which increased GC apoptosis and led to a larger number of atretic follicles. Fu et al. [73] found that IGF-1 secreted by MSCs may induce overexpression of Bcl-2 in GCs and therefore increase their resistance to chemotherapy. Babitha et al. [47] found that IGF-1 can lead to a dose-dependent decline in Bax expression in GCs. HGF has numerous biological effects, including stem cell maintenance, antiapoptosis, antifibrosis, anti-inflammation, and promoting angiogenesis [74]. Several studies have strongly supported the antiapoptotic effects of HGF primarily by inducing the expression of antiapoptotic proteins, such as Bcl-2 [75]. Liu et al. [76] found that HGF treatment is related to an increase in Bcl-2 and a decrease in Bax expression. Furthermore, VEGF, is an effective mitogen for vascular endothelial cells and a critical growth factor for angiogenesis, which is able to induce angiogenesis and form new vessels in vivo [77]. Antiapoptotic or cytoprotective activities of VEGF in GCs have also been demonstrated in previous studies [78]. Therefore, improving local endogenous VEGF expression in the ovary is beneficial to ovarian angiogenesis, GC survival, and follicular growth [56, 79]. Previous studies have shown that FGF2, IGF-1, and HGF have cytoprotective and antiapoptotic activities, which can stimulate endogenous VEGF expression in GCs [47]. In our study, the results also demonstrated that hAD-MSC-CM containing growth factors, as extracellular viability factors, improved the local microenvironment in ovarian tissue affected by POI, leading to decreased Bax expression and increased Bcl-2 and endogenous VEGF expression in ovarian cells, which inhibit chemotherapy-induced GC apoptosis, promote angiogenesis, and regulate follicular development in injured ovaries affected by POI. Therefore, we speculate that these growth factors might inhibit chemotherapy-induced GC apoptosis, promote angiogenesis, and regulate follicular development partly by upregulating Bcl-2 expression, downregulating Bax expression, and promoting local VEGF expression in the ovaries of POI rats. Moreover, a previous study has shown that Bax expression was lower and the E2 secretion level was higher in GCs treated with VEGF and FGF2 compared to either VEGF or FGF2 alone, which indicates that these growth factors might function in a synergistic manner to inhibit GC apoptosis and promote angiogenesis in ovaries [71].

## Conclusions

In conclusion, this study demonstrates that hAD-MSC transplantation can reduce ovarian injury and improve ovarian function in rats with POI. hAD-MSC transplantation improves ovarian function in rats with POI at least partly through a paracrine mechanism. The presence of a paracrine mechanism accounting for hAD-MSC-mediated recovery of ovarian function in chemotherapy-induced POI might be attributed to the growth factors secreted by hAD-MSCs, which inhibit chemotherapy-induced GC apoptosis, promote angiogenesis, and regulate follicular development by independent mechanisms and/or by upregulating Bcl-2 expression, downregulating Bax expression, and promoting local endogenous VEGF expression in the ovaries of POI rats. However, detailed mechanisms need to be investigated in subsequent experiments. This study provides evidence of the protective effect of hAD-MSCs and hAD-MSC secretome in chemotherapy-induced POI, which might provide new potential therapeutic strategies for patients with POI in the future.

## Abbreviations

CTX: Cyclophosphamide
FSH: Follicle-stimulating hormone
GC: Granulosa cell
hAD-MSC: Human amnion-derived mesenchymal stem cell
POI: Premature ovarian insufficiency
MSC: Mesenchymal stem cell
FGF2: Fibroblast growth factor 2
IGF-1: Insulin-like growth factor-1
VEGF: Vascular endothelial growth factor
HGF: Hepatocyte growth factor
G-CSF: Granulocyte-colony stimulating factor
DAPI: 2-(4-Amidinophenyl)-6-indolecarbamidine dihydrochloride
CCK-8: Cell Counting Kit-8
SD: Sprague-Dawley
AMH: Anti-Müllerian hormone
ANOVA: Analysis of variance
AYAs: Adolescents and young adults
CM: Conditioned media
L-DMEM: Dulbecco’s modified Eagle’s medium low-glucose
FBS: Fetal bovine serum
E2: Estradiol
FSHR: Follicle-stimulating hormone receptor
BSA: Bovine serum albumin
KGF: Keratinocyte growth factor
MVIS: Median value of immunoreactivity score
HPFs: High-power fields
DAB: 3,3′-Diaminobenzidine
HE: Hematoxylin and eosin
